# Joint analysis of transcriptional and post- transcriptional brain tumor data: searching for emergent properties of cellular systems

**DOI:** 10.1186/1471-2105-12-86

**Published:** 2011-03-30

**Authors:** Raffaele Fronza, Michele Tramonti, William R Atchley, Christine Nardini

**Affiliations:** 1 Key Laboratory of Computational Biology, MPG-CAS PICB, Shanghai, PR China; 2 Biocomputing Unit, University of Bologna, Bologna, Italy; 3 DEIS, University of Bologna, Bologna, Italy; 4 Department of Genetics, North Carolina State University, Raleigh, NC, USA

## Abstract

**Background:**

Advances in biotechnology offer a fast growing variety of high-throughput data for screening molecular activities of genomic, transcriptional, post-transcriptional and translational observations. However, to date, most computational and algorithmic efforts have been directed at mining data from each of these molecular *levels *(genomic, transcriptional, etc.) separately. In view of the rapid advances in technology (new generation sequencing, high-throughput proteomics) it is important to address the problem of analyzing these data as a whole, i.e. preserving the emergent properties that appear in the cellular system when all molecular levels are interacting. We analyzed one of the (currently) few datasets that provide both transcriptional and post-transcriptional data of the same samples to investigate the possibility to extract more information, using a joint analysis approach.

**Results:**

We use Factor Analysis coupled with pre-established knowledge as a theoretical base to achieve this goal. Our intention is to identify structures that contain information from both mRNAs and miRNAs, and that can explain the complexity of the data. Despite the small sample available, we can show that this approach permits identification of meaningful structures, in particular two polycistronic miRNA genes related to transcriptional activity and likely to be relevant in the discrimination between gliosarcomas and other brain tumors.

**Conclusions:**

This suggests the need to develop methodologies to simultaneously mine information from different levels of biological organization, rather than linking separate analyses performed in parallel.

## Background

Currently, it is possible to observe the activity (over-, under- expression, presence or absence of mutations) of almost all molecules of a given type (mRNA, miRNA, DNA) in a single screen using high-density chips [1], or sequencing related techniques [2,3]. Lately, the number of studies using microarray platforms for analysis of mRNA are quickly being followed by similar analyses related to miRNAs [4,5]. Only recently both types of variables were analyzed simultaneously [6-8], while, typically, both types of data are analyzed in search for (i) molecules sharing similarity, using simply the expression available at the time (*unsupervised *approaches, [9]) e.g. clustering [10,11] and association networks [12-14] or (ii) similarity with -or dependency from- other types of traits, providing for example clinical classes or other non-molecular information on the samples (*supervised *approaches, [9]) i.e. Significant Analysis of Microarray (SAM [15]), Gene Set Enrichment Analysis (GSEA [16]). However, this approach implies to analyze separately different aspects of a system (e.g., transcriptional and/or post-transcriptional mechanisms) and the results may not be concordant with analyses of the system as a whole. For example, interactions among miRNAs and mRNAs may be underestimated or completely overlooked. This lack of information can be expressed as missing the *emergent *properties of the system. While the concept of emergent properties is well known in Systems Theory, it has only recently become an important concept in the area of life sciences, thanks to the relatively new approach of Systems Biology [17-20]. Emergent properties arise from hierarchical integration of the individual components and organizational levels of complex systems, and, biologically, they are only manifest when the organism is considered in its entirety. Analogous to emergent properties in systems biology is the concept of latent variables in multivariate statistics. Latent variables are so-called hidden variables generated in certain types of multivariate analysis (e.g. factor analysis, see below) which are not evident in original observed data. Rather, these latent variables emerge from consideration of the covariance patterns when a large number of relevant variables are analyzed simultaneously. These latent variables may reflect a summarization of causal indicators underlying observed biological variability. Given the parallelism between biological systems' emergent properties and latent variables, we sought- quite naturally- to investigate the ability of latent variables to describe emergent properties, by applying multivariate analysis simultaneously to different parts of a biological system, and notably to transcriptional and post-transcriptional data. Previously, successful parallel multi-platform analyses were performed integrating genomic and transcriptional level, by using CGH arrays or SNPs and cDNA arrays [21,22]. This approach portend to explain variations observed at the transcriptional level, based on information at the genomic level. These approaches can annotate and map different types of probe IDs onto genomic coordinates [23], or add analyses at the translational level [24]. However, to date, simultaneous analysis of miRNA and mRNA from the same tissue have used only profile correlations [6]. Herein, we expand analyses of molecular covariation beyond correlation of expression profiles by using the multivariate statistical procedure of multiple or common Factor Analysis (FA, [25]). This procedure is widely used to reduce the dimensionality of multivariate data and to do so in a manner that elucidates the underlying or latent structure of the observed variation. Succinctly speaking, for a given set of molecular data, factor analysis partitions the observed pair-wise correlations between variables into that molecular covariation that is common between the variables from that which is unique to the individual variables. Application of FA directly on biological data without any *a priori *hypothesis about latent variables is ideal for data reduction. With this approach FA was used extensively to cluster microarray data [26-28]. The use of the *a priori *knowledge on how each sample maps on tumor classes to constrain the relation between the latent variables under study and the factors obtained permits further data interpretation. In other words we perform a FA that is driven by data (hypothesis) pre-established to find latent variables that could be investigated to obtain biological information [29]. To constrain the factor model we used Linear Discriminant Analysis (LDA, [25]), a technique used to classify a set of observations into categories (a dichotomy in our case). In particular, in the following we will describe the methodology and the results obtained from applying FA to mRNA and miRNA data simultaneously, with the goal to identify information that is not obvious when the analysis is performed on the 2 datasets separately, or when using other approaches. In particular, the identification of a set of co-localized miRNAs (cluster) with possible relevance for the molecular description of gliosarcomas, appears to emerge from this analysis only, showing the potential FA in the identification of emergent properties. Besides LDA, other classifiers (Support Vector Machine, Naive Bayes, Neural Network and k-Nearest-Neighbours) were also tested and performances are listed in Table S9 of the Additional file 1. We only briefly mention here that most of the performances are identical for all the classifiers, and only for the Glioblastomas discrimination LDA shows slightly more accuracy. These results indicate that the classification analysis is robust and gives stable results independently from the choice of the classification algorithm. Factor analysis proceeds from a matrix of pair-wise correlations to extract a small number of *factors *that describe the major patterns of common covariation. More formally, the common factor model is based on the equation *D *= *LF *+ *E*, where *D *are the observed variables, *L *are the common factors, *F *are the coefficients or scores of the factors and *E *are the unique factors, under the assumptions that the unique factors are uncorrelated whith each other and that *F *and *E *are independent. Since only common variation is analyzed, these individual factors describe the latent structure underlying the major patterns of molecular covariation. The sign and magnitude of the factors coefficients reflect the extent and direction of the correlation between each variable and individual factor and describe the relative contribution of each variable to a particular pattern of multivariate changes. FA derives a set of *factor scores *that gives the relative location of each item in the reduced latent variable subspace. The resultant factors, coefficients and scores are interpreted in light of biological knowledge about the specific data under study. FA can define a biological model about the underlying nature of molecular covariation (e.g. number of patterns of covarying elements and their relative importance). These models are evaluated both biologically and statistically and subsequently used to explain the structure and dynamics of complex biological systems. FA and Principal Component Analysis (PCA, [30]) involve several of the same statistical components and are both useful for data reduction. Therefore few words on the rationale for choosing FA instead of PCA are necessary. PCA is an exact mathematical method that returns a single solution where each component is orthogonal and represents an element of variance in the samples (both biological and non-biological). Therefore, although it is possible to force PCA in order to relax constraints like orthogonality we chose to apply FA since it is more a natural choice to analyzes the common or shared molecular variations and thus, describe the patterns of biological variation. Besides, the method commonly used to estimate the common or shared molecular variations are based on multiple regression and therefore, for most of the applications of FA, this standard approach is stable. There exist several approaches to perform data reduction and classification (see for example Bayesian classifiers [31-33], Support Vector Machine [34], K-nearest-neighbor [35]), however, FA has already been used successfully in various applications related to molecular biology, like the identification of multidimensional patterns of molecular covariation able to describe proteins' structures [36]. More classical approaches have been designed for effective clustering in the analysis of cDNA microarrays and Expressed Sequences Tag (ESTs) [37], as well as in specific applications to identify genes and pathways related to biological categories that could be associated to relevant phenotypes in both yeast and humans [38] or to test and validate hypotheses on the association of gene expression to cisplatin resistance in ovarian cancer cell lines [39]. One of the advantages of this approach over hierarchical clustering is the possibility to include genes in more than one category. More recently, FA was used to filter informative and non-informative data from microarray for gene expression [40]. Variations of classical FA (Bayesian factor analysis) have been used to identify the latent structure that describes the relationship between transcription factors and genes, using microarray data [41]. Previously, this approach was used to perform gene network reconstruction in *E. Coli *taking advantage of literature information, DNA sequences and expression arrays [42]. We now propose to apply FA to the composite analysis of multilevel molecular data.

## Results and Discussion

Because miRNAs and mRNAs are processed together, from now on, Factors will always be likely to include both mRNAs and miRNAs in their composition. To avoid confusion on the meaning of the word *gene*, we use the term *coding genes *to refer to mRNAs and the generic term *genes *to refer both to mRNAs and miRNAs. The interpretation of factors based on associating them to mRNAs/miRNAs (separately considering positive/negative scores) is a novelty of the presented approach, and will be discussed in details in the coming sections. In particular, in the following we will describe: how we identified the latent factors and we will give their interpretation, both using mRNA and miRNA (indirect) functionalities. Then, we will describe the biological structure emerging from this analyis, and we will speculate on its clinical meaning. Finally, we offer a comparison with the results of an analysis done in parallel, although more comparisons are provided in the Additional file 1.

### Identification of Multilevel Latent Structures

We performed several Factor Analyses and obtained Models characterized by 1 to 5 factors (named here Model *n*, *n *= 1,..., 5). We have used Kaiser criterion [43] to identify the number of factors that show a large variance (common variance in each factor greater than a given threshold, *t*) and therefore carry a large amount of the information hidden in the data. Given *t *= 1 the number of information-rich factors appears to be 4. Therefore, FA was performed with a growing number of such factors, from the one with higher variance, up to 5, to test the appropriateness of the variance threshold. We then confirmed the validity of a subset of the Models using LDA to identify which factor (or linear combination of factors) was able to best classify tumor grade and histopathology, based on the statistical significance of Fisher exact test [44]. This test, suited for contingency tables where one or more expected frequencies are below 5, evaluates the null hypothesis associated with LDA that there are no statistically significant differences between the *a priori *clinically defined groups. The models for which the null hypothesis was rejected were retained (see Table 1 and Methods for details). Therefore, we performed 4 LDA, namely between a class and its complement: i.e. high/low grade, anaplastic/non-anaplastic, glioblastoma/non-glioblastoma and gliosarcoma/non-gliosarcoma, following the original classification in [6]. We did not consider oligodendroglioma relevant, because of a single sample available. Model 3 appears to be the most suitable, since it is able to discriminate between anaplastic and non-anaplastic tumors with 100% accuracy (based only on Factor 2) and the other two types of tumors with ≃ 92% accuracy. Since anaplastic tumors are low grade tumors, Factor 2 is relevant in the identification of low grade tumors in general with ≃ 92% accuracy, since the only oligodendroglioma appears to be elusive. It is worth noting that Model 4 shows the same performance scores, but with a greater number of factors and Factor 4 does not appear to be involved in class identification.

**Table 1:** Model Selection - Discriminant Analysis

Model	Tumor Grade	Anaplastic	Glioblastoma	Gliosarcoma
1	-	-	-	-
2	F 2 (**0.92**, *0.045*)	F 2 (**1**, *0.015*)	-	-
3	F 2 (**0.92**, *0.045*)	F 2 (**1**, *0.015*)	F 1+F 2 (**0.92**, *0.015*)	F 1+F 3 (**0.92**, *0.018*)
4	F 2 (**0.92**, *0.045*)	F 2 (**1**, *0.0015*)	F 1+F 2 (**0.92**, *0.015*)	F 1 (**0.92**, *0.018*)
5	F 1 (**0.92**, *0.045*)	F 1 (**1**, *0.0015*)	-	F 5 (**0.92**, *0.018*)

Tumors type and grade dual discrimination. In bold **Accuracy**; in italic *p-value*.

### Interpretation of Multilevel Latent Structures mRNA

#### Functional Analysis

Working solely on Model 3, the mRNAs in each factor were processed to detect enriched Gene Ontology (GO, [45]) terms or UniProt (SP, [46]) keywords. The magnitude and sign of the factor scores (not the factor coefficients from the eigenvectors) give their relative relationship with the expression of miRNA and mRNA. Consequently, each row in the 3 factors score matrix (*F*1, *F*2 and *F*3) was split into positive and negative portions (*F*1^+ ^and *F*1^−^; *F*2^+ ^and *F*2^−^; *F*3^+ ^and *F*3^−^) and analyzed separately. *F*1^+ ^is associated with GO terms related to response to stress and external stimuli. Terms from SP keywords like *secreted *and *glycoprotein *were also found in this subset. Thus this factor appears then to be related with cell functions that process signal from the external environment to the cell with membrane receptors involved to the signal transduction. *F*2^− ^is also involved in the signaling, including categories related to cell adhesion, it appears then to be related to functions like chemotaxis that are involved in inflammation processes. Finally, *F*3^+ ^contains coding genes that are related to the biological process that goes under the general term *gene expression*. Gene expression includes all the mechanisms such as transcription, translation, RNA maturation, proteins transport and ubiquitination by which information coded in the DNA is converted to a functional product. All results are summarized in Table 2.

**Table 2:** Functional Analysis

Factor	Ontology Terms	Ontology
*F*1^+^	Response to external stimulus	GO_-_BP
	Secreted, glycoprotein	SP
	Plasma Membrane, transucer, extracellular, receptor	GO_-_MF, GO_-_CC, SP
*F*2^-^	Signal, glycoprotein	SP
	Cell Adhesion	SP
	Extracellular region	GO_-_CC
*F*3^+^	Gene Expression	GO_-_BP

Functional analysis of the factors in Model 3. GO: Gene Ontology; BP: Biological Process; CC: Cellular Component; MF: Molecular Function; SP: Swiss Prot.

#### miRNA *Indirect *Functional Analysis

Since miRNAs are not included in any ontology database, we performed an indirect functional analysis by screening the functional terms associated with the experimentally validated target coding genes of the miRNAs, extracted from TarBase [47]. Once the target coding genes were identified, they were manually annotated via GO terms or SP keywords, as above (see Table 3).

**Table 3:** Indirect Functional Analysis

	*F*3^+^			*F*2^-^	
**miRNA**	**Target Gene**	**Terms**	**miRNA**	**Target Gene**	**Terms**

hsa-miR-9	BACE1	*Endoplasmic reticulum, Golgi apparatus, integral to membrane, Gol, gi apparatus, Aspartyl protease*	hsa-miR-422b	-	
hsa-miR-363*	-		hsa-miR-23a	CXCL12	CELL ADHESION, CHEMOTAXIS, POSITIVE REGULATION OF MONOCYTE CHEMOTAXIS, EXTRACELLULAR SPACE
hsa-miR-20b*	ARID4B	TRANSCRIPTION REGULATION, NUCLEUS	hsa-miR-193a	-	
	MYLIP	PROTEIN UBIQUITINATION, UBIQUITIN-PROTEIN LIGASE ACTIVITY, NERVOUS SYSTEM DEVELOPMENT	hsa-miR-155	AGTR1	REGULATION OF NATRIURESIS, REGULATION OF CELL GROWTH, POSITIVE REGULATION OF INFLAMMATORY RESPONSE, REGULATION OF BLOOD VESSEL SIZE BY RENIN-ANGIOTENSIN
	HIPK3	TRANSCRIPTION REGULATION, NUCLEUS		LDOC1	*negative regulation of cell proliferation*
	CDKN1A	*negative regulation of cell proliferation, response to toxin, response to UV, positive regulation of programmed cell death, cyclin-dependent protein kinase inhibitor activity*		MATR3	*nuclear matrix, RNA binding, protein binding*
hsa-miR-19a*	PTEN	*induction of apoptosis, regulation of cyclin-dependent protein kinase activity*		BACH1	*Transcription regulation, Nucleus, transcription factor activity*
hsa-miR-17-5p*	E2F1	TRANSCRIPTION REGULATION, NUCLEUS, TRANSCRIPTION FACTOR ACTIVITY		TM6SF1	MEMBRANE
	NCOA3	POSITIVE REGULATION OF TRANSCRIPTION, DNA-DEPENDENT, NUCLEUS, HISTONE ACETYLTRANSFERASE ACTIVITY		TP53INP1	INDUCTION OF APOPTOSIS
hsa-miR-17-3p*	-	-			
hsa-miR-130b	-	-			

Target coding genes and annotation terms for miRNAs that were selected in Model 3. In capital letters categories that are related with the ones found by *direct *enrichment analysis on mRNAs. In italics categories not shared with the *direct *enrichment analysis. For *F*3^+ ^miRNAs marked with * belong to the identified polycistronic miRNA genes.

#### mRNa/miRNA Complex Functional Annotation

We then checked the functional classification's coherence between the indirect and direct functional analysis, within each significantly annotated factor (i.e. *F*2^−^, *F*3^+^, since no miRNA appeared in *F*1^+^). Thus, globally speaking, *F*1^+ ^annotation is unchanged and related to functions that are responsible for signal transduction. In *F*2^−^, 3 out of 7 target coding genes (CXCL12, TM6SF1 and AGTR1) are annotated with terms that can be associated to the categories significantly varied in the mRNA functional analysis: *F*2^− ^is then confirmed to be a factor involved in functions related with adhesion and/or chemotaxis. For the miRNAs in *F*3^+^, 5 out of 8 target coding genes (ARID4B, MYLIP, HIPK3, E2F1 and NCOA3) are functionally related with the *gene expression *term found in the mRNA functional analysis. Interestingly, most of the terms (4/5) are related with mechanisms of transcription regulation and only one with protein ubiquitination. After direct and indirect annotation, 2 miRNAs and 31 human coding genes in *F*3^+ ^were selected as belonging to the same category (see Additional file 1, Table S5). Not surprisingly, most of the coding genes in this list are not predicted to be targets of the 2 miRNAs that appear in the factor. In fact, the biological meaning of the result is a set of genetic elements that share covariability in the expression pattern and we know that, e.g. in animals, most of the control on gene expression is performed by tuning translation. Therefore, the levels of miRNAs and the mRNAs of direct targets are not directly correlated [48]. As it is also suggested in [6] we can imagine that our list of coding genes contains the possible subset of indirect targets (functionally related with the regulation of the transcription) of two miRNAs: miR-17-5p, and miR-20b. Globally, *F*3^+ ^is confirmed to be associated with gene expression, with transcription regulation being the most common mechanism of expression.

#### Emergent Properties

**Figure 1:**
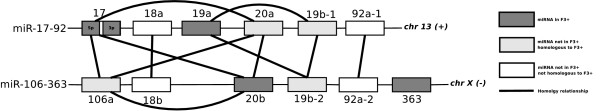
**Organization of miRNA**. *clusters *miR-17-92 and miR-106-363. Structure of the two polycistronic miRNA gene and the relations between miRNAs.

Since the transcription regulation term (*F*3^+^) appears to give the clearest biological information, coherent in mRNAs and miRNA, we focused our efforts on this part of the analysis. The total sets of mRNAs and miRNAs returned from this analysis are listed in Table S6 and S7 of the Additional file 1. *Latent Structure Chromosomal Localization: *Most of the miRNAs in *F*3^+ ^belong to two polycistronic miRNA genes where miRNAs are lying in close proximity on the chromosome. (The named *clusters *are given in italics throughout the paper to improve readability and avoid confusion with clusters emerging from supervised or functional analyses). These polycistronic miRNA genes are involved in cell proliferation, apoptosis suppression, tumor angiogenesis [49] and T cell leukemia [50]. The first polycistronic gene (miR-17-92) is composed by 7 miRNAs and maps on Chromosome 13 whereas the second one (miR-106-363) maps on Chromosome × and contains 6 miRNAs, details are shown in Figure 1. The two *clusters *are closely related, in fact, each miRNA on one *cluster *has at least one homologous in the other *cluster *except for miR-17-3p and miR-363 that do not share homology with the other miRNAs (shown in Figure 1). As further corroborating test, we observed that, when searching the target coding genes of homologous miRNAs (miR-20a, miR-17-5p and miR-106a) the list of predicted targets (Targetscan, [51]) is identical for all miRNAs. Moreover, we notice that only two homologous groups of miRNAs in the *cluster *(miR-18 and miR-92) are not part of *F*3^+^. If we look at their sequence in detail we observe that they are very similar to miR-20a with only two mismatches: one in the loop (miR-18a and 18b) and one after the supplementary pairing region (miR-18b). This can represent a partial functional redundancy since all the known key regions in target recognition are identical. Conversely, miR-92 does not share any significant homology with the other members of the *cluster *(except for the seed region with miR-363). Taking into consideration all the redundancies in the *clusters*, most of the transcript targets in *F*3^+ ^are probably under the regulation effect of the expressed miRNAs. It is worth noting that a cross-hybridization effect in miRNAs could be considered the mechanism responsible for these association in *clusters*. But, as reported by the authors of the dataset [6], each primer and probe contained zip-coded sequences specifically assigned to each miRNA to increase the specificity of each reaction so that even small differences in miRNA were amplified and detected. So, this artifact can be discarded as explanation for the emerging of *clusters *of miRNA. *Statistical Relevance: *Interestingly, in *F*3^+^, only 2 miRNAs (hsa-mir-9 and hsa-mir-130b) out of 7 do *not *belong to any of these two *clusters*. Their role was shown respectively to be related to the molecular pathogenesis of ovarian cancer [52] as well as to schizophrenia and Human T-cell leukemia Virus-1 (HTLV-1) transformation [53,54]. Six more miRNAs (miR-106a, miR-18a and miR-18b, miR-20a, miR19b-1 and miR-19b-2) that belong to these two *clusters *could not be part of our analysis, as they were not part of Liu's original dataset. Given the high density of miRNAs in these *clusters*, we used the hypergeometric distribution to compute the probability associated with the hypothesis that a random sampling would give the same result in terms of number of *cluster *members in *cluster *miR-17-92 (3 members out of 4 total), in *cluster *miR-106-363 (2 members out of 3 total) and in both (5 members out of 7 total). The reference group for computing the probability consists of the total number of detected miRNAs (93). The resultant probabilities were Bonferroni corrected and were equal to 3.6 × 10^−3^, 0.045 and 2.3 × 10^−7 ^respectively. All three are statistically significant.

### Speculations on Molecular-Clinical Implications

Ultimately, we speculated on how the two *clusters *that emerge in *F*3^+ ^can, along with the molecular analysis performed on *F*1, discriminate between gliosarcomas and non-gliosarcomas. This choice is due to the fact that our analysis has shown that the combination of factors that carry the more coherent functional information (both from miRNAs and mRNAs signals) was the combination able to discriminate glioscarcomas from other tumors. Believing that such a coherence could hide strong biological meanings we focused on gliosarcomas the efforts to detect emergent properties. This complex task, that cannot be fully explained with the data and results in hand, can take advantage of intriguing observations emerging from the analysis. We notice, in fact, that the presence of the sarcomatous element, that derives from an endothelial hyperplasic lesion [55], is a characteristic of these kinds of tumor. The hyperplasic lesion is a proliferation of vessel-wall components that contains endothelial cells, myofibroblast, smooth muscle cells and other components of the vascular endothelium [56]. In [49] it is also shown that *cluster *miR-17-92 is related to solid tumors angiogenesis. The finding of this *cluster*, and the homologous miR-106-363, in the factor that contributes to discriminate gliosarcomas, could then indicate an involvement in the development of the sarcomatous element.

### Identification and Interpretation of Simple Latent Structures

In this Section we present results obtained from analyzing with FA and LDA the two datasets (mRNA and miRNA) separately. Our original hypothesis dealt with the ability of the complex analysis to identify emergent properties. To evaluate this hypothesis we produced a 3 factor model with factor analysis on the two expression matrices separately. Next, we analyzed the two series of factor scores using separate LDA. In this Section we identify with *F_mi_i *Factor *i *obtained from the miRNA dataset and with *F_m_j *Factor *j *from the mRNA dataset (*Fk *continues to identify Factor *k *from the joint dataset. Regarding the identification of the latent structures, as expected and given the larger size of the mRNA matrix, the results in terms of discrimination power among tumor classes and the functional analysis are unchanged. However, the situation is different for the miRNA data. As shown in Table 4 only high/low grade tumors and anaplastic/non analplastic categories are predicted with the same accuracy (and on the same factor, *F_mi_*2). The accuracy is lower, 0.83 (*p *= 0.08) versus 0.92 (*p *= 0.015) for the glioblastoma/non-glioblastoma category. This occurs because one of the glioblastomas is predicted as a non-glioblastoma. Furthermore, the discrimination appears to be based on a linear model composed only by *F_mi_*1 and not on a combination (see *F*1 and *F*2 in the complex analysis). The discrimination between gliosarcomas and its dual class is the worst, as accuracy drops to 0.75 (*p *= 0.23) and *F_mi_*3 is not used in discrimination. For what concerns the interpretation of the latent structures, out of the 18 miRNAs selected, 9 are in common with the joint analysis and 9 represent a new set of miRNAs. Five of the miRNAs in the new set are associated with biological terms, and only one (hsa-miR-126) is shared by more than one factor (*F_mi_*1 and *F_mi_*2). *F_mi_*1 contains 5 terms, *F_mi_*2 2 terms (a subset of *F_mi_*1) and *F_mi_*3 2 terms (for details see Additional file 1, Table S8). These are related with the regulation of the transcription (in *F_mi_*1 and *F_mi_*3) and they show some overlap with the mRNAs Factors annotation. Namely, biological terms in *F_mi_*1 overlap with all the three *F_m _*whereas terms in *F_mi_*2 overlap only with *F_m_*2. Terms in *F_mi_*3 are found both in *F_m_*2 and *F_m_*3. With respect to the comparison to the complex analysis, since these miRNAs are mostly clustered in homologous factors it is possible to associate *F_mi_*3 with *F*1, *F_mi_*2 with *F*2 and *F_mi_*3 with *F*1). The miRNAs shared with the *complex *analysis and that return an annotation are in *F_mi_*2 (both miR-155 and miR-23a) and *F_mi_*3 (miR-155). However, without the joint analysis there is no obvious rationale to associate miRNA factors with mRNA factors. This is because, crucially, the 18 miRNAs obtained are distribuited over factors that are decoupled from the factors returned from the *simple *mRNA data analysis. Therefore this approach does not suggest any obvious association between the two sets of factors. As a consequence, the interpretation of this latter (*simple*) analysis is limited to the indirect functional annotation of this small set of miRNA (Additional file 1, Table S8). Therefore, the activation of the polycistronic clusters miR-17-92 and miR-106-363 does not emerge when miRNAs are analysed separately. In summary, combining the two datasets and applying FA and LDA, provides an obvious way to associate the translational and post-translational information. In particular, although the mRNA latent structure is the same in the simple and complex analysis, and consequently the functional annotation is the same, hidden signals present in the smaller dataset (miRNA set) appear to be amplified by the signals present in the larger dataset (mRNA set) thanks to their association in a common latent structure.

**Table 4:** Performances of Model 3 using only miRNA data.

(a) Tumor Grade			(b) Anaplastic		
	
	**High/Low Grade**			**Anaplastic**	
	**P High**	**P Low**		**P Anap**	P *Anap
	
High	**9**	**0**	Anap	**10**	**0**
Low	**1**	**2**	* Anap	**0**	**2**
	
	**p = 0.045**			**p = 0.015**	
	
(c) Glioblastoma			(d) Gliosarcoma		
	
	**GlioblastEoma**			**Gliosarcoma**	
	**P Gilo**	P*GLio		**P Gsar**	P * Gsar
	
Glio	**5**	**1**	Gsar	**2**	**1**
* Glio	**1**	**5**	* Gsar	**2**	**7**
	
	p = 0.08			p = 0.23	

These Tables shows the classification performances of Model 3 on expression data of miRNA only. Significant classifications in bold (*p *< 0.05). Anap: Anaplastic; *Anap: non Anaplastic, Glio: glioblastoma; *Glio: non glioblastoma, Gsar: gliosarcoma; *Gsar: non gliosarcoma.

## Conclusions

The capability to discriminate between *a priori *defined classes can be achieved in a variety of ways (a comparison with supervised and unsupervised algorithms is provided in the Additional file 1). However, the capacity to generate factors explaining the complexity of the molecular interactions requires the ability to construct multilevel clusters. With the data at hand we showed that this cannot be achieved in parallel analysis (versus simultaneous or joint) of the two datasets (mRNA and miRNA) or with other approaches we evaluated. The interpretation of factors based on associating them to mRNA/miRNAs represents the major contribution of this work. Certainly, the study of [6] shows sample size limitations (12 patients enrolled) therefore our analyses must be considered as an exemplar of the factor analysis approach. Globally, based on this analysis, since the miRNAs in *F*3^+ ^belong to two redundant *clusters *of miRNA, we can speculate that: 1) one of the biological functions in which these *clusters *could be involved is the regulation of the transcription and 2) in some way, in brain tumors these two *clusters *are active whereas, in normal cells, only miR-17-92 appears to be constitutively expressed. Probably both *clusters *act on the same set of coding genes, but the two loci are regulated separately in normal cells [50]. Nevertheless, despite this strong relationship between the 2 *clusters *it is difficult to understand how this redundancy works effectively in cells. However, the finding of a possible activation of the polycistronic genes miR-17-92 and miR-106-363 represents an encouraging evidence that the factorization of the miRNA and mRNA data can reveal latent structure in the configuration of the expression levels in tumor samples. Despite obvious limitations, we believe our results clearly show that this approach is a very powerful one for the study of multilevel *omic *data, which in turn can bring more insight into understanding the complex mechanisms of the transmission of information in the cell as a whole.

## Methods

**Figure 2:**
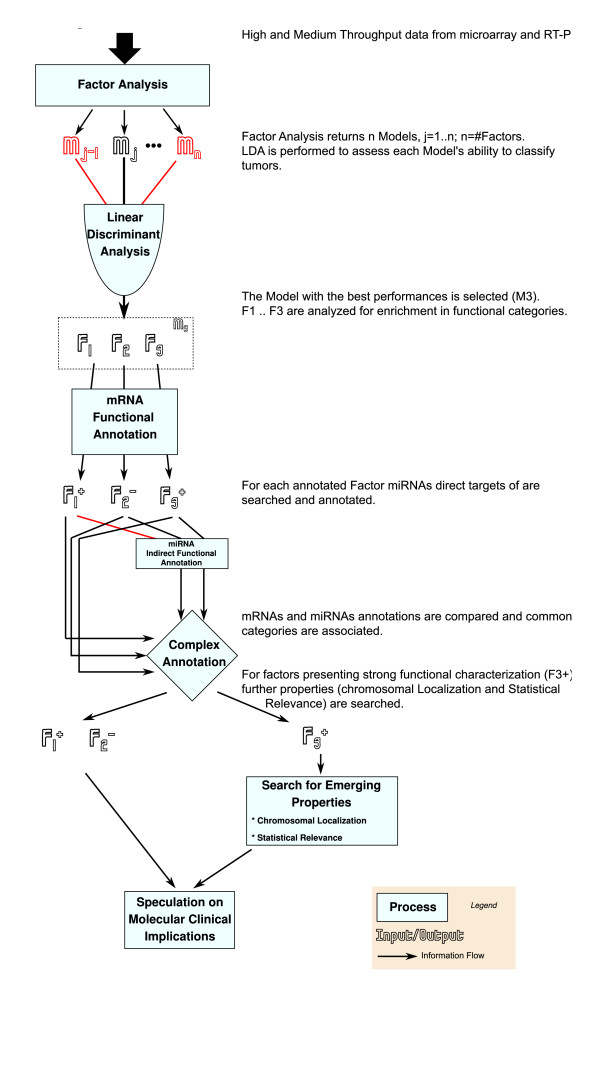
**Schematic view of the *complex *analysis performed jointly on mRNA and miRNAs from the same 12 tumor samples**.

In this work, we applied FA to the dataset from [6]. These data consist of 12 microarray samples (for mRNA genome-wide expression, around 14,500 coding genes) and 12 real-time PCR (for the profile of 93 miRNAs), performed on the same 12 human primary brain tumor biopsies (details in Additional file 1, Table S1). On this test case dataset, we first identified the best FA model (i.e. the appropriate number of factors) based on the models' ability to explain the relevant clinical and histopathological information. Next, we characterized the factors based on 3 properties: 1) their ability to discriminate among tumor types -this was done using Linear Discriminant Analysis (LDA, [25]), a supervised classifier able to find the linear combination of factors which best separates two pre-defined classes; 2) their functional biological characterization with the help of literature and databases; 3) their complex biological characterization, by searching novel properties emerging from the joint analysis of miRNA and mRNAs. The procedure is summarized in Figure 2.

### Data Preprocessing

Data from [6] were transformed by computing log_2 _of the intensity value of mRNA expression (miRNA data come already in log_2 _from real-time PCR). Quality selection filtering was performed removing every row (mRNA or miRNA expression across 12 experiments) with maximum fold change below 2.5; this reduced the dataset from 7182 IDs to 4966 IDs. The filtering was decided to select genetic elements with strong signal of variation. This criterion was selected as natural consequence of the filtering performed by the authors of the dataset [6] that used the same conditions to reduce the number of the IDs. Data were also normalized in different ways according to:

• , were *M_i _*and *m_i _*are the maximum and minimum values in the *i*th row, and *x_ij _*is the expression of gene *i *on sample *j*.

• , where *μ_i_*, is the average expression level in the *i*th row, and *x_ij _*is the expression of gene *i *on sample *j*.

The two methods map the expression level in an interval comprised between 0 and 1 the first and *μ_i _*and *μ_i _*+ 1 the second (in order to introduce in the model also the difference in expression beween genes). The two normalizations give identical results in the Factor Analysis step as expected. In fact, expression signals obtained from qPCR are different from signals obtained from microarrays due to the extended dynamic range of the former. It is common [57,58], in order to validate a set of coding genes obtained by microarray, to express the mRNA level in each sample as a fraction of the expression level in the sample in which that mRNA is most abundant. So, from this point on, miRNA and mRNA expression data were analyzed together, as a single expression table with normalization .

### Factor Analysis

The Factor Analysis model can be defined in matrix notation as: *D *= *LF *+ *ε*, where *D*(*m *× *n*) represents the data matrix, *L*(*m *× *l*) is the factors *loadings *matrix, *F*(*l *× *n*) is the factors *scores *matrix and *ε*(*m *× *n*) is the *unique factors *matrix. Furthermore, *m *are the number of samples, *n *the number of genetic elements and *l *the number of factors. Our model assumes that *F *and *ε *are indipendent, *E*(*F*) = 0, and *Cov*(*F*) = *I*. Under these conditions *Cov*(*D*) = *LL^T ^*+ *Cov*(*ε*), for the sake of clarity *LL^T ^*is named *communality *and *Cov*(*ε*) *uniqueness*. Variability in a human tumor expression dataset arises from several sources besides tumor type, including human variability (sex, age, race) and experimental variability (systematic and stochastic errors). Available information is about tumor types, therefore, our model explicitly involves tumor types variability, and groups other causes within the *ε *term, showing the power of the FA method. In our work, we were interested in discovering the hidden or latent structure within tumor types, therefore FA is applied using the model *D *= *X^T^*. The R-package HDMD developed by Lisa McFerrin at North Carolina State University was used to take advantage of the principal axes algorithm. Communalities were estimated by iteratively updating the diagonal of the correlation matrix and solving the eigenvector decomposition. Axes were rotated to simple structure using the Promax algorithm to improve their interpretability. The simple structure obtained after rotation meets the requirements proposed by Thurstone [59,60] to ensure the stability of FA results. The factor score matrix was analyzed for each of the 5 models (from 1 to 5 embedded factors). The scores associated to the genes within each factor were ranked in descending order. All 3 factors presented a similar scores distribution with average *μ *≃ 0 and standard deviation *σ *≃ 0.75. Selection has been performed by looking at the value distribution of each row of matrix F and then considering as genes associated with a factor only those whose corresponding score is outside the 2*σ *interval. In this way, only genes with a strong relation in the same factor were selected.

### Discriminant Analysis

The factor *loadings *coefficients matrix of each model was used to perform LDA. Four dichotomous categories (given by a class and its negate, e.g. glioblastoma/non-glioblastoma etc.) were defined (Table 1). LDA was also performed to assess the most likely class of sample T18 which had an ambiguous classification (glioblastoma/gliosarcoma), see Additional file 1, Table S2. R-package *MASS *[61], function *lda() *configured to perform a classical cross-validation classification (jack-knife method, also known as *leave-one-out *validation) was used. In particular we used a *step-wise greedy *strategy, i.e. checking performances with one factor, and adding another factor, iteratively. All possible equivalent combination of factors were tested, and the most performant with the smallest number of factors involved was chosen.

### Model Selection

To evaluate the performances of each factor model on the four tumor classes, we evaluated the contingency table obtained from the discriminant analysis by Fisher's exact test. The null hypothesis assuming that the discrimination between two tumor classes is due to chance was rejected for *p *< 0.05. For models with similar prediction scores we kept the one with fewer factors.

### Functional Classification

On both FA and clustering (used as alternative method to our approach, see Additional file 1) functional analysis was performed using the online tool DAVID [62,63] using GO terms, Kegg pathways terms, SP keywords and features and InterPro terms. The whole list of 4876 probe ID was used as background population. In order to reduce the number of non significant associations, a resulting functional cluster was further analyzed if and only if it contained at least one category with Benjamin score < 0.05. The indirect functional analysis performed to describe miRNAs relevance was performed by searching manually in TarBase [47] all the known coding genes that are target of the miRNAs identified by the FA and clustering. Then for each gene a list with all the associated GO terms was compiled. Due to the small number of targets obtained no *p*-value could be associated to any GO term.

## Authors' contributions

RF analyzed the data with the help of MT and provided the biological interpretation. WRA provided strong theoretical support for the study, CN ideated the study and wrote the paper with the contribution of WRA and RF. All authors have read and approved the final manuscript.

## Supplementary Material

Additional file 1 **Supplementary information**. Click here for file
