# Supplementary material for: Joint analysis of transcriptional and post- transcriptional brain tumor data: searching for emergent properties of cellular systems
Source: BMC Bioinformatics. 2011 Mar 30;12:86. doi: 10.1186/1471-2105-12-86 (PMC3078861; doi:10.1186/1471-2105-12-86)
Supplement: Additional file 1 — Supplementary information. [file 1471-2105-12-86-S1.PDF]

Joint Analysis of Transcriptional and post- Transcriptional Data: Searching Emergent Properties in Brain Tumor Samples Supplementary Information

Table 1. Tumor Types and Categories

Sample	Tumor Type	C.1 (low/high)	C.2 (anapl/non-anapl)	C.3 (gliob/non-gliob)	C.4 (glios/non-glios)
T19	Oligodendroglioma	l	n	ngb	ngs
T21	Anaplastic mixed glioma	l	a	ngb	ngs
T24	Anaplastic mixed glioma	l	a	ngb	ngs
T18	Glioblastoma/Gliosarcoma	h	n	ngb/gb	ngs/gs
T20	Glioblastoma	h	n	gb	ngs
T22	Glioblastoma	h	n	gb	ngs
T23	Glioblastoma	h	n	gb	ngs
T25	Glioblastoma	h	n	gb	ngs
T26	Glioblastoma	h	n	gb	ngs
T27	Glioblastoma	h	n	gb	ngs
T28	Gliosarcoma	h	n	ngb	gs
T29	Gliosarcoma	h	n	ngb	gs

Schematic summary of tumors types used in the experiments, and category used for the discriminant analysis

Labeling of Sample T18 gliosarcoma/glioblastoma

Particular care must be taken with respect to sample T18, which comes in [1] with an ambiguous classification of *glioblastoma/gliosarcoma*. Gliosarcomas are variants of glioblastomas where the sarcomatous element is mixed with the gliomatous.

For this reason we defined T18 as gliosarcoma, as this appears to add specificity to the glioblastoma histopathological classification of the sample.

However, to disambiguate its definition, and to assess the importance of this classification in the final results on Model 3, we assigned T18 to both categories and their complement (Gliosarcoma/Non Gliosarcoma; Glioblastoma/Non Glioblastoma) and then performed the LDA on the 4 different datasets obtained.

If LDA is able to correctly discriminate the known classes, we infer it can also properly assign this ambiguous sample. As shown in Table 2 c) and Table 2 d), LDA performs a better discrimination when T18 is assigned to

the Gliosarcoma class rather than to Glioblastoma. When the sample was assigned to the category of the Non Gliosarcoma, LDA lost completely the capacity to detect gliosarcomas in the samples. Conversely, when T18 was assigned to the Glioblastoma class, LDA assigned 9 samples instead of 7 to Glioblastoma.

Table 2. Discriminant Analysis on Sample T18.

(a) Tumor Grade

	**High/Low Grade**	
	**P High**	**P Low**
High	**9**	**0**
Low	**1**	**2**
	**p=0.021**	

(b) Anaplastic

	**Anaplastic/*Anaplastic**	
	**P Anap**	**P *Anap**
Anap	**10**	**0**
* Anap	**0**	**2**
	**p=0.004**	

(c) Glioblastoma

	**Glioblastoma**			
	P Glio	P *Glio	**P Glio**	**P *Glio**
Glio	7	0	**6**	**0**
* Glio	2	3	**1**	**5**
	T18 as Glio		**T18 as *Glio**	
	p = 0.072		**p=0.012**	

(d) Gliosarcoma

	**Gliosarcoma**			
	**P Gsar**	**P * Gsar**	P Gsar	P * Gsar
Gsar	**3**	**0**	0	2
* Gsar	**1**	**8**	0	10
	**T18 as Gsar**		T18 as * Gsar	
	**p=0.02**		p = 1	

These tables show the classification performances of Model 3 when T18 is classified in 4 different possible ways: Gliosarcoma and dual, and Glioblastoma and dual. Significant classifications ($p < 0.05$ after Bonferroni correction) are shown in bold. Classification performances are statistically significant when T18 is classified as Gliosarcoma and not as Glioblastoma. Anap: Anaplastic; *Anap: non Anaplastic, Glio: glioblastoma; *Glio: not glioblastoma, Gsar: gliosarcoma; *Gsar: not gliosarcoma.

Alternative Approaches

In order to compare our approach to other likely tools, we choose to analyze the mRNA/miRNA dataset with 3 methods.

First we used hierarchical clustering [2] an unsupervised and extremely popular tool for microarray analysis.

Given the poor results obtained, we sought to preprocess the dataset with SAM [3], another very popular, but supervised method that imposes a structure in the data according to an *a priori* classification of the dataset (i.e. grade and tumor type), to favor the identification of elements related to the clinical classification.

Clustering has been successfully used in the past to classify clinical samples based on microarray data, therefore the current analysis aims at showing more the inability of this approach to identify emerging properties (in this case the polycistronic miRNA clusters) rather than its limited ability to classify samples.

Finally, we compared the results with the method proposed in [1].

Cluster Analysis

R was used to perform clustering on the whole dataset based on euclidean distance and correlation between each couple of experiments, obtaining two sets of $n(n-1)/2 - n = 120$ distances.

Sample clusters identified two low grade tumours (T21 and T24) clustered together whereas the third low grade oligodendroglioma (T19) was clustered with two high grade glioblastoma (T20 and T26). The high grade sample glioblastoma/gliosarcoma (T18) was clustered with a gliosarcoma (T28). Two subclusters group together samples T22 and T27 and samples T23 and T25 (glioblastoma), respectively. All of these sub-clusters were combined by the clustering procedure in three clusters: T22, T27, T29, T18 and T28 were assigned to cluster 1; T23 and T25 to cluster 2 ; T19, T20, T26, T21 and T24 to cluster 3 (see Figure 1(a)).

In samples clustering we followed the empirical approach that the number of clusters should be close to $\sqrt{n}$, with n indicating the number of

items to be clustered.

Observing the clusters composition, a latent structure in the tumor samples can be hypothesized. To validate this hypothesis, we used χ^2 statistical test to verify if one of the three clusters contained information on one of the four tumor types. However, no cluster was able to discriminate significantly ($p > 0.05$) between tumor classes.

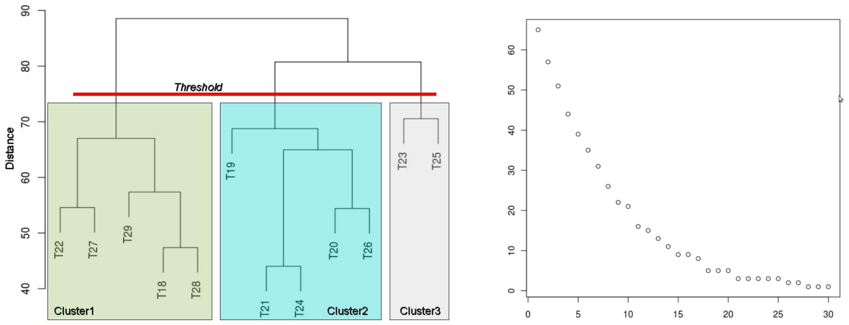

(a) Sample Clusters (b) Clusters size

Figure 1. Results of Clustering. Figure 1(a) shows the sample clusters obtained without preliminary SAM analysis. Results are identical for the analysis performed after SAM analysis. Figure 1(b) shows the variability in the clusters number around threshold values varied in the interval [0.4-3] for SAM + Clustering analysis.

Table 3. Contingency Tables for tumours groups discrimination from Cluster Analysis (with and without preliminary SAM Analysis)

	Cluster 1		Cluster 2		Cluster 3	
Low	5	4	3	0	3	0
High	0	3	7	2	2	7
Anap	2	5	2	0	2	0
Not Anap	5	0	8	2	3	7
Glioblast	4	2	6	0	3	2
Not Gliobast	3	3	4	2	2	5
GlioSarc	3	0	3	0	5	4
Not GlioSarc	2	7	7	2	0	3

Cluster 1:T22,T27,T29,T18,T28. Cluster 2:T23,T25. Cluster 3:T21,T24,T19,T20,T26. Anap: anaplastic; Glioblast: glioblastoma; GlioScac: gliosarcoma.

SAM + Cluster Analysis

R package samr was used to perform a reduction of the dataset on the basis of the tumors grade (High/Low) and the tool was run with $\Delta = 0.3$.

SAM basically works as a generalized t-test to identify genes whose average behavior in one class is statistically significantly different from the one in other(s) class(es).

With these settings, SAM returned a subset of 419 (8.5%) genetical components, of which 413 (8.5%) were Affimetrix ID and 6 (6.7%) miRNAs.

The analysis performed on the set reduced by SAM, lead to the same sample clustering obtained without SAM pre-processing. Distance between the first three samples (T19, T21, T24), the low grade tumours, is similar to the distance found with clustering alone. Therefore, even after imposing a structure in the datasets, none of the clusters can discriminate among classes.

It is worth noting that Anaplastic/Non Anaplastic tumours can be identified (with and without the use of SAM) only if the dendrogram is cut in order to isolate the subcluster T21, T24. But this approach leads to 7 and 10 clusters with and without SAM preprocessing, respectively, which, starting from 12 items defeats the purpose of clustering (in samples clustering we followed the empirical approach that the number of clusters should be close to $\sqrt{n}$, with n indicating the number of items to be clustered).

Cutoff threshold for identifying genes clusters was set to 2.1, after visual inspection of a diminished variability in the clusters number around this value (Figure 1(b)). This lead to the identification of 5 clusters composed respectively by 141 (2 miRNA), 164 (1 miRNA), 34 (1 miRNA), 48 (0 miRNA) and 32 (2 miRNA) elements. These clusters were analyzed to verify if they were significantly enriched in any GO functional category. No functional terms were found statistically significant.

Comparison with the Original Work

Comparing our work with [1] is not straightforward, since the purpose of the authors was to detect couples of miRNA-mRNA using the correlation

coefficient as measure of targeting of the mRNAs by miRNAs. Our aim is different, we want to aggregate mRNA and miRNA that are the manifestation of underlying biological phenomena.

Limitation using correlation [4], mutual information [5] and other simple measures of covariability when the variables under study are miRNA and mRNA have been briefly illustrated above, and consist of the fact that most of the direct targets of a miRNA (especially in animals) do not share the same profile, since the mechanism that is used to control the expression of a target is inhibition of the translation, and therefore transcription of the target remains untouched.

Factor analysis is better suited, since it allows the extraction of covariance patterns that reveal underlying genetic structure related to *shared* covariability.

However, in our analysis, other source of noise can persist, due, for example, to our ignorance about some latent structure in the samples, like sex or age. Given this information different models could be selected. For instance, the model with 4 factors reveals that there are two factors that explain some shared variance in our samples that are not related with tumor types. Our weak control on sample characteristics cannot help us to use this model to make hypothesis on factor structure.

Liu and coworkers tried to identify the proper range of correlation values able to identify direct/indirect targets and elements located in nearby areas on the genome. Overall, our analysis highlighted 19 miRNA starting from 93 whereas [1] found 30 relevant miRNA from high correlating couples.

Only 9 miRNA are shared between the two list (see Table 4).

Only two miRNAs (miR-19a and miR-9) from $F3^+$ are reported in Liu's list. Their analysis focuses on couples detection and no evidence of miR-17-92 or miR-106-363 clusters emerges.

Alternative Classification Analysis

Loading scores contain a signal that a classifier can extract to discriminate between tumor classes. We used 4 standard alternative classifiers:

Support Vector Machines, Neural Networks, Naïve Bayes and K-Nearest Neighbour downloaded from http://www.patternrecognition.co.za/sourcecode.html. We used the default parameters to classify the four tumor classes. The performances of each classifier are shown in Table 9. From these results it is clear that the selection of the classifier is not a critical step of the analysis (except maybe for the Naive Bayes that gives poor performances for the Glioblastoma class) and that other supervised techniques could be introduced in the pipeline to constrain the factor model.

Table 4. Comparision between miRNA found in this work and in Liu [1]

miRNA in Model 3	miRNA in Liu work
hsa-miR-95	+
hsa-miR-346	-
hsa-miR-340	+
hsa-miR-181b	-
hsa-let-7b	+
hsa-miR-9	+
hsa-miR-363	-
hsa-miR-20b	-
hsa-miR-19a	+
hsa-miR-17-5p	-
hsa-miR-17-3p	-
hsa-miR-130b	-
hsa-miR-99a	+
hsa-miR-194	+
hsa-miR-422b	-
hsa-miR-23a	-
hsa-miR-193a	+
hsa-miR-155	+
hsa-miR-200c	-

Projection of the set of miRNAs selected in Model 3 on the list of miRNAs selected in the original work from where data were obtained ([1]).9 miRNAs (out of 19) are common (+) between our list and Liu's list; 10 miRNAs are present only in our list (-).

References

1. Liu T, Papagiannakopoulos T, Puskar K, Qi S, Santiago F, et al. (2007) Detection of a microrna signal in an in vivo expression set of mrnas. PLoS One 2: e804.

2. Eisen MB, Spellman PT, Brown PO, Botstein D (1998) Cluster analysis and display of genome-wide expression patterns. Proc Natl Acad Sci 95: 14863-14868.

3. Tusher VG, Tibshirani R, Chu G (2001) Significance analysis of microarrays applied to the ionizing radiation response. Proc Natl Acad Sci 98: 5116-5121.

4. RRSokal, FJRohlf (2003) Biometry. New York: Freeman.

Table 5. $F3^+$: miRNA and mRNA annotated with Transcription Regulation term

miRNA	Gene Name	Gene Description
	AOF2	amine oxidase (flavin containing) domain 2
	ASXL1	kiaa0978 protein
	C19ORF2	chromosome 19 open reading frame 2
	CHD7	chromodomain helicase dna binding protein 7
	CHD8	chromodomain helicase dna binding protein 8
	ETV1	ets variant gene 1
	GTF2H3	general transcription factor iih, polypeptide 3, 34kda
	HEY1	hairy/enhancer-of-split related with yrpw motif 1
	HIC2	hypermethylated in cancer 2
	ID4	inhibitor of dna binding 4, dominant negative helix-loop-helix protein
	KCNH2	potassium voltage-gated channel, subfamily h (eag-related), member 2
	MAZ	myc-associated zinc finger protein (purine-binding transcription factor)
hsa-miR-17-5p	NONO	non-pou domain containing, octamer-binding
hsa-miR-20b	OLIG2	oligodendrocyte lineage transcription factor 2
	POLR2E	polymerase (rna) ii (dna directed) polypeptide e, 25kda
	PPP2R1A	protein phosphatase 2 (formerly 2a), regulatory subunit a (pr 65), alpha isoform
	PRMT5	protein arginine methyltransferase 5
	RBBP4	retinoblastoma binding protein 4
	RBPJ	recombining binding protein suppressor of hairless (drosophila)
	RERE	arginine-glutamic acid dipeptide (re) repeats
	RTF1	rtf1, paf1/rna polymerase ii complex component, homolog (s. cerevisiae)
	SOX12	sry (sex determining region y)-box 12
	SSRP1	structure specific recognition protein 1
	STAT5B	signal transducer and activator of transcription 5b
	TBL1XR1	transducin (beta)-like 1x-linked receptor 1
	TCF12	transcription factor 12 (htf4, helix-loop-helix transcription factors 4)
	TCF3	transcription factor 3 (e2a immunoglobulin enhancer binding factors e12/e47)
	TCF7L1	transcription factor 7-like 1 (t-cell specific, hmg-box)
	YBX1	y box binding protein 1
	ZNF195	zinc finger protein 195
	ZNF43	zinc finger protein 43 (htf6)

Table 6. Number of mRNA found in Factors after multilevel anaysis

Factor	# mRNA
F1	332
F2	421
F3	368

5. Cover TM, Thomas JA (2001) Elements of Information Theory. John Wiley and Sons.

Table 7. Number of miRNA found in Factors after multilevel anaysis

Factor	# miRNA
F1	3
F2	9
F3	8

Table 8. miRNA from Simple Structure Analysis. Functional Annotation

miRNA	Annotation	Factor
hsa-miR-106b	response to gamma radiation	F1
hsa-miR-29b	cell fate determination, positive regulation of gene expression	F1
hsa-miR-23a	*cell adhesion, chemotaxis, positive regulation of*	
	monocyte chemotaxis, extracellular space	*F2*
hsa-miR-126	membrane to membrane docking, cell junction	F1,F2
hsa-miR-155	*regulation of natriuresis, regulation of cell growth*	
	positive regulation of inflammatory response, regulation of blood vessel size	
	by renin-angiotensin, Transcription regulation, Nucleus	
	negative regulation of cell proliferation, negative regulation of cell proliferation	
	response to toxin, response to UV, positive regulation of programmed cell death	
	cyclin-dependent protein kinase inhibitor activity	
	nuclear matrix RNA binding, protein binding	*F2,F3*
hsa-miR-210	cell-cell signaling	F3
hsa-miR-27a	regulation of transcription, DNA-dependent	F3

Only miRNAs that give an annotation are listed in the table. $F_{mi}1$: Factor 1; $F_{mi}2$: Factor 2; $F_{mi}3$: Factor 3. *Italics*: miRNAs and annotations shared with the Complex Analysis.

Table 9. Model Selection - Alternative Classification Analysis

Model	Tumor Grade	Anaplastic	Glioblastoma	Gliosarcoma
SVM	(**0.92**, *0.045*)	(**1**, *0.0015*)	(**0.83**, *0.08*)	(**0.83**, *0.045*)
NB	(**0.92**, *0.045*)	(**1**, *0.0015*)	(**0.58**, *1*)	(**0.83**, *0.077*)
NN	(**0.92**, *0.045*)	(**1**, *0.0015*)	(**0.83**, *0.06*)	(**0.83**, *0.045*)
kNN	(**0.92**, *0.045*)	(**1**, *0.0015*)	(**0.83**, *0.08*)	(**0.92**, *0.018*)

Tumors type and grade dual discrimination. In bold **Accuracy**; in italic *p-value*. SVM: Support Vector Machine; NB: Naive Bayes; NN: Neural Network; kNN: k-Nearest-Neighbours.
